# Selection of a Novel DNA Aptamer for Assay of Intracellular Interferon-Gamma

**DOI:** 10.1371/journal.pone.0098214

**Published:** 2014-05-21

**Authors:** Beibei Cao, Yan Hu, Jinhong Duan, Jie Ma, Danke Xu, Xian-Da Yang

**Affiliations:** 1 Institute of Basic Medical Sciences, Chinese Academy of Medical Sciences & Peking Union Medical College, Beijing, China; 2 Cancer Institute & Hospital, Chinese Academy of Medical Sciences & Peking Union Medical College, Beijing, China; 3 School of Chemistry and Chemical Engineering, Nanjing University, Nanjing, China; University of Houston, United States of America

## Abstract

Interferon-gamma (IFN-γ) is a glycoprotein generated by lymphocytes that possesses anti-tumor, antiviral and immunomodulatory functions. IFN-γ assays are broadly employed in immunological research and clinical diagnostic tests. Intracellular IFN-γ staining, in particular, is an important immune assay that allows simultaneous determination of cellular phenotype and antigen-specific T cell response. Aptamers have great potential for molecule detection and can bind to target molecules with high affinity and specificity. In this study, a novel 59-mer DNA aptamer (B1–4) was developed for assay of intracellular IFN-γ. The selected aptamer bound to IFN-γ with a Kd of 74.5 nM, with minimal cross-reactivity to albumin. The aptamer was also found capable of binding with paraformaldehyde-fixed IFN-γ. Moreover, B1–4 could enter permeated and paraformaldehyde-fixed lymphocytes, and bound to intracellular IFN-γ produced by these cells. When FITC-labeled B1–4 was used to stain a group of lymphocytes, the average fluorescence of the cells was positively correlated with the number of PMA-stimulated lymphocytes within the group. A standard curve could thus be established for assessing the fraction of IFN-γ-producing cells in a cluster of lymphocytes. The selected aptamer hence provides a novel approach for assaying intracellular IFN-γ generated by a group of lymphocytes, and may have application potential in both scientific research and clinical laboratory test.

## Introduction

Assays of IFN-γ are commonly used in both immunological research and clinical diagnosis. IFN-γ is predominantly generated by a number of immune cell types including CD8+T cells, dendritic cells, activated CD4+T cells, natural killer (NK) cells, and other lymphocytes [1], [2]. It is a glycoprotein that possesses antitumor, antiviral, anti-proliferative and immunomodulatory functions [1]. IFN-γ is an important inflammatory cytokine in response to various pathogens [1]. The level of IFN-γ expression largely reflects the status of cellular immunity and provides diagnostic information for various infectious diseases. IFN-γ is detected in the circulating blood of patients with HIV infection or autoimmune diseases such as systemic lupus erythematosus [3]. Current IFN-γ detection methods are based on specific IFN-γ antibodies, and include enzyme-linked immunosorbent assay (ELISA) [4]–[6], enzyme-linked immunospot assay (ELISPOT) [7], [8], and intracellular cytokine staining (ICS) [9], [10]. Among these methods, ICS is a commonly used important assay for assessing T cell reactivity [11]–[13], since it is the only immunological technique that can simultaneously determine both the cellular phenotype and the antigen-specific T cell response. Moreover, ICS assay is reasonably fast and can generate result a few hours after drawing the blood. Notably, the major reagents used in ICS are fluorescent dyes, which are safer than radioactive compounds such as ^3^H-thymidine or ^51^Cr.

While conventional antibody-based cytokine immunoassays are well developed, they are still hindered by the relatively high cost associated with large-scale measurement [14], [15]. To develop new method as a substitute for antibody-based immunoassay, research efforts have been focused on aptamer-based method for cytokine detection [16]. Aptamers are small single-stranded nucleic acids (DNA or RNA) that can fold into intricate three-dimensional structures and recognize a wide variety of targets, including peptides, proteins, or many other compounds [17], [18]. Similar to antibodies, aptamers have high-binding affinity and specificity for its targets [19]–[21]. As an emerging class of ligand for target recognition, aptamers provide several advantages over antibodies, such as low molecular weight, low-immunogenicity, easy modification, and limited synthesis cost. Furthermore, aptamers are more resistant to high temperature than protein, and can retain their structure after repeated cycles of denaturation/renaturation [22]. Because aptamers are more robust than antibodies, aptasensors can be regenerated and used multiple times. Based on these advantages, aptamers have been broadly employed to construct new diagnostic tools and biosensing probes; for example, Yang et al developed a light-switching aptamer probe for quantitative detection of PDGF [23]; Tombelli and colleagues built an aptamer-mediated biosensor for quartz crystal microbalance (QCM) and surface plasmon resonance (SPR) to assay HIV-1 Tat protein [24].

IFN-γ aptamer has been used as probes for detection of extracellular IFN-γ [25]–[28]. So far, however, there is no report on using aptamer for assay of intracellular IFN-γ. In the present study, we explored whether aptamer could be applied to stain the intracellular IFN-γ produced by lymphocytes. To achieve this goal, we developed a novel DNA aptamer (B1–4) that could enter permeated lymphocytes and bind to both natural and paraformaldehyde-fixed IFN-γ. When FITC-labeled B1-4 was used to stain a group of lymphocytes, the average fluorescence of the cells was positively correlated with the number of IFN-γ-producing lymphocytes within the sample. The results indicated that novel methods for measuring intracellular IFN-γ might be developed based on this newly identified DNA aptamer.

## Materials and Methods

### Ethics statement

Human peripheral bloods were collected after obtaining written informed consent from the donors. The procedure was approved by the Ethics Committee at Chinese Academy of Medical Sciences and Peking Union Medical College.

### Reagents

Oligonucleotide primers were synthesized by Invitrogen (Shanghai China). Recombinant Human IFN-γ of at least 95% purity was purchased from SinoBio (Shanghai China). Bovine serum albumin (BSA) was purchased from TBD Science (Tianjin China). Monodispersed magnetic urea-formaldehyde microspheres were purchased from Baseline Chromtech (Tianjin China). Trypsin was purchased from Amresco (US). Streptavidin-coated magnetic beads were purchased from Promega (USA). 1-Ethyl-3-(3-dimethyllaminopropyl)-carbodiimide hydrochloride (EDC), phorbol 12-muristate 13-acetate (PMA), and ionomycin were purchased from Sigma (US). Aptamers A2 (5′TGCCCGTGTCCCGAGGAGGTGCCCTATTTTGCTTGATTATCTCTAAGGGATTTGGGCGG3′) and T2 (5′GGGGTTGGTTGTGTTGGGTGTTGTGT3′) were synthesized by Invitrogen (China).

### Immobilization of target on magnetic beads

The target for aptamer selection is recombinant human IFN-γ of at least 95% purity. The conjugation of the target to carboxylatedmagnetic beads was accomplished via cross-linking of carboxyl and amine groups. The carboxylated UF beads (1×10^5^, 200 µL) were washed twice with 200 µL of PBS. The beads were resuspended in 200 µL deionized water dissolving with IFN-γ protein (0.5 µg) and incubated with 200 µL EDC (40 mM) at room temperature with gentle stirring for 2 h. The magnetic beads were then washed for three times with PBS, and stored at 4°C. The same method was employed to conjugate the beads with other substances, including paraformaldehyde-fixed IFN-γ protein and BSA, for specificity assays of the selected aptamer.

### SELEX library and primers

A starting ssDNA library consisting of 59-mer oligonucleotides with central 21-mer long randomized sequences was synthesized. The library sequence was 5′-TGCGTGTGTAGTGTGTCTG (N21) CTCTTAGGGATTTGGGCGG-3′, where N represented a randomized nucleotide of either A, G, C or T. An FITC-labeled 5′ primer (5′- TGCGTGTGTAGTGTGTCTG-3′) and a biotin-labeled 3′ primer (5′-CCGCCCAAATCCCTAAGAG-3′) were used in the PCR for the synthesis of double-stranded DNA molecules, which was then mixed with streptavidin-coated magnetic beads for 20 min at room temperature. After denaturing in alkaline condition (0.1 M NaOH), the FITC-conjugated single-stranded DNA (ssDNA) was separated from the mixture by applying a magnetic field, and used for aptamer selection.

### In vitro SELEX process

Salmon sperm DNA (0.1 mg/mL) and BSA (1 mg/mL) were added to the binding buffer (PBS) to reduce the background interference. The procedures of selection were as follows: in the first selection round, the ssDNA pool (200 pmol) was first heated at 95°C for 5 min and then cooled to 0°C for 15 min immediately. The IFN-γ coated beads were suspended in 200 µL of binding buffer containing 200 pmol of random ssDNA. After incubation with the mixture at 37°C for 30 min with gentle shaking, the unbound oligonucleotides were removed by washing three times with 500 µL of binding buffer. Subsequently, target-bound oligonucleotides on beads were amplified by PCR with FITC- or biotin-labeled primers (25 cycles of 95°C 30 s, 56°C 30 s, 72°C 40 s, the Taq polymerase and dNTPs were obtained from Genestar). The PCR amplified dsDNA were separated from the biotinylated antisense ssDNA strand by streptavidin-coated magnetic beads and used for the next round of SELEX. After several rounds selection, the selected ssDNA pool was amplified by PCR using unmodified primers and cloned into *Escherichia Coli* with the TA cloning kit for DNA sequencing.

### Flow cytometric analysis

To monitor the enrichment of each selection pool, the FITC-labeled ssDNA pool was incubated with IFN-γ-coated magnetic beads in 0.2 ml of selection buffer containing 10% FBS at room temperature for 30 min. The beads were washed twice with 0.5 ml of binding buffer, suspended in 0.2 ml of binding buffer, and evaluated with flow cytometry (Accuri C6 Flow Cytometer, BD). The FITC-labeled random ssDNA was used as control.

To evaluate the binding specificity of aptamers to target, FITC-labeled aptamer was incubated with IFN-γ, paraformaldehyde-fixed IFN-γ, trypsin, IgG or BSA coated magnetic beads, respectively. The beads were washed twice with 0.5 ml of binding buffer, suspended in 0.2 ml of binding buffer, and analyzed by flow cytometry.

To evaluate the binding affinity of aptamers to IFN-γ, we incubated IFN-γ-coated beads and blank beads with various concentrations of FITC-labeled aptamer B1–4 in binding buffer at room temperature for 30 min. The beads were washed twice with binding buffer, suspended in binding buffer, and analyzed by flow-cytometry. To compensate the influence of the bead-bound aptamers, the mean fluorescence of aptamer bound to the IFN-γ-coated beads was used to calculate the Kd after subtracting the fluorescence generated by the aptamers bound to the blank beads [29]. The equilibrium dissociation constant (Kd) was obtained by fitting the dependence of fluorescence intensity of specific binding on the concentration of the aptamers to the equation: Y = B max X/(Kd+X), where Y represented the reciprocal of the average fluorescence intensity, X represented the reciprocal of aptamer's concentration, and Bmax represented the maximum binding capacity of aptamer bound to IFN-γ.

### Preparation of human peripheral blood mononuclear cells

Venous blood was collected from healthy volunteers. Peripheral blood mononuclear cells (PBMC) were obtained by Ficoll-Paque density gradient centrifugation. All donors were required to sign an informed consent form according to procedures approved by the Ethics Committee at Chinese Academy of Medical Sciences and Peking Union Medical College.

To obtain PBMCs from 20 ml of human blood, we usually layered approximately 7 ml of whole undiluted blood on 7 ml of Ficoll-Paque in three 15 mL conical polycarbonate tubes. Tubes were centrifuged at 1500 rpm for 20 min with high acceleration and no brake. Layers made up of red blood cells/platelets, Ficoll-Paque, a fluffy thin disc of PBMCs and plasma (from bottom to top) should be visible. The PBMC ring was carefully aspirated and put into a 50 mL tube. The latter was then filled with RPMI and centrifuged at 1500 rpm. The cell pellet obtained was washed again with PBS. PBMCs were then resuspended in complete medium at 4–6*10^6^ cells/ml and 0.5 ml were aliquoted in Falcon conical polystyrene tubes. The PBMC were stimulated with PMA and ionomycin for 6 h [30], in the presence of brefeldin A[31], [32]. Samples incubated with BFA alone served as the nonstimulated controls.

After stimulation, the cells were washing three times with 500 µL of PBS, and 1 ml of paraformaldehyde was added while constantly vortexing at high speed. The cells were incubated in fix solution for 20 min on ice, washed in PBS twice, and stored at 4°C overnight. For staining, fixed cells were pelleted, vortexed and resuspended in 100 µl of PBS. Permeabilization was achieved by washing samples in perm solution once. Alternatively, saponin could be added to samples to a final concentration of 0.5%. Aptamer B1–4, aptamer A2, antibody, or isotype were added to each tube, vortexed and incubated in the dark for 2 h. Cells were washed once with perm buffer and resuspended in 500 µl PBS, and subjected to flow cytometry analysis.

### Statistics

Statistical analysis was performed using SPSS software. All data were presented as a mean value with its standard deviation (mean ±SD).

## Results

### Aptamer selection

**Figure 1:**
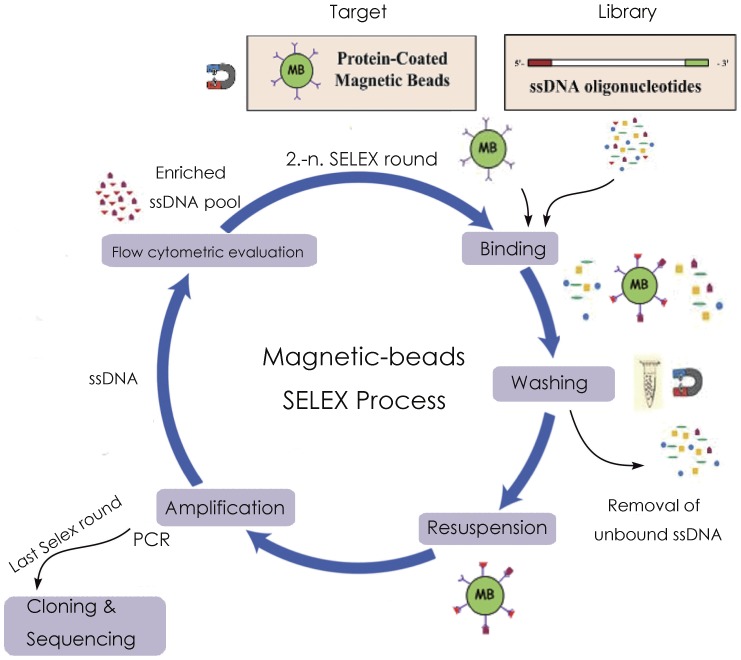
Schematic illustration of DNA aptamer selection by SELEX. In short, a 200-γ-coated magnetic beads at room temperature for 30 min. Salmon sperm DNA (0.1 mg/mL) and BSA (1 mg/mL) were used to reduce nonspecific binding. After washing, bound ssDNA on magnetic beads were eluted by heating at 95°C for 5 min, and subjected to PCR amplification with fluorescent tag to start the next cycle. After several cycles of selection, the enriched pool of ssDNA was cloned and sequenced for the identification of the individual aptamer.

**Figure 2:**
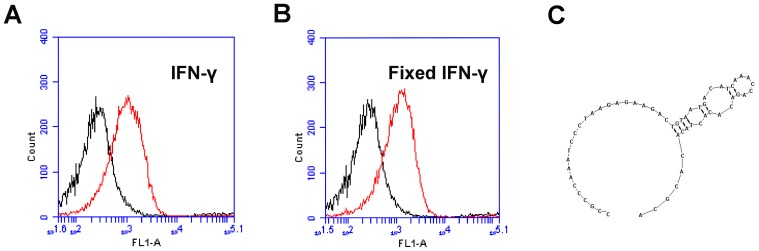
Flow cytometry monitoring of the enrichment of aptamers. Compared with the initial random DNA pool (black curves), flow cytometry revealed an increase in fluorescence intensity of ssDNAs bound to the IFN-γ protein (A) and paraformaldehyde-fixed IFN-γ protein (B) after the last round of selection (the red curves). (C) The predicted secondary structure of B1–4.

To generate high affinity IFN-γ aptamers in this study, a standard SELEX technique was employed using recombinant IFN-γ protein and paraformaldehyde fixed IFN-γ as the targets. The details of the selection process were shown in Figure 1. IFN-γ or paraformaldehyde fixed IFN-γ was conjugated covalently to carboxylated magnetic beads using EDC as catalyst. Natural and fixed IFN-γ were used in turn as the targets in alternative rounds of selection. To monitor the enrichment of IFN-γ-binding DNAs during each round of selection, the beads were incubated with FITC-labeled ssDNA pool and subjected to flow cytometry analysis. Compared with the random DNA library pool, increasing fluorescent intensities were observed with the progress of selection, suggesting that more ssDNA bound to the beads coated with IFN-γ or paraformaldehyde-fixed IFN-γ (Figure 2). The enriched IFN-γ-binding DNAs were subsequently cloned, and 96 clones were analyzed for further characterization. Among these clones, one aptamer termed B1–4 showed relatively good binding to both natural and paraformaldehyde-fixed IFN-γ. The DNA sequence of the aptamer B1–4 was 5′-CCGCCCAAATCCCTAAGAGAAGACTGTAATGACATCAAACCAGACACACTACACACGCA-3′. The predicted secondary structure of B1-4 was shown in Figure 2C.

### Binding properties of aptamer B1–4

**Figure 3:**
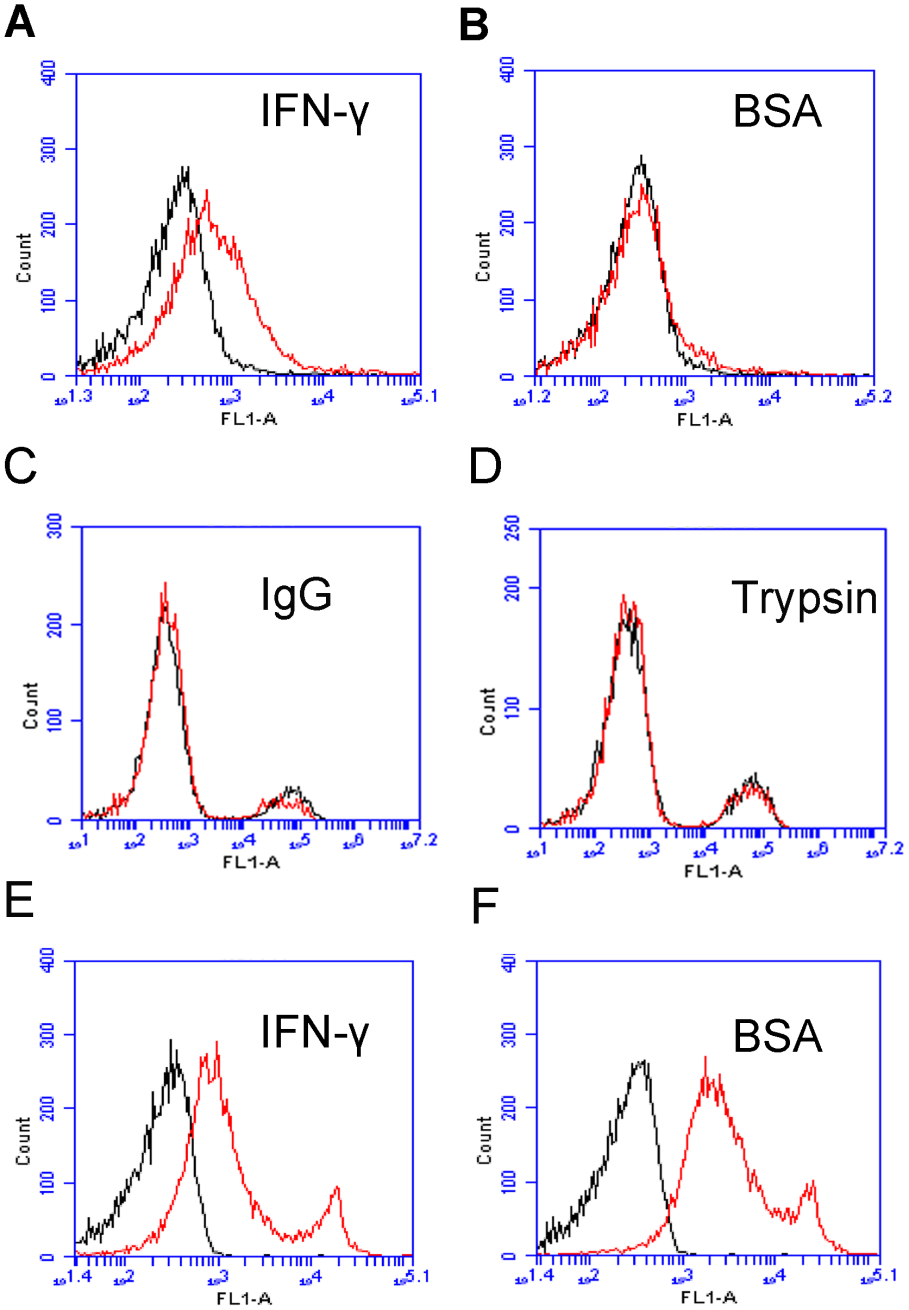
Flow cytometry assessment of the bindings to IFN-γ or BSA by FITC-labeled aptamers B1–4 and T2. (A) IFN-γ beads treated with FITC-labeled B1–4. (B) BSA beads treated with FITC-labeled B1–4. (C) IgG beads treated with FITC-labeled B1–4. (D) Trypsin beads treated with FITC-labeled B1–4. (E) IFN-γ beads treated with FITC-labeled T2. (F) BSA beads treated with FITC-labeled T2. Black lines represent the control fluorescent signals generated by FITC-labeled random DNA from the library pool.

Specificity is one of the most important characteristics of aptamer performance. Since albumin is the most abundant protein in blood, we examined the binding of the aptamer B1–4 to albumin. In addition, we also assessed the aptamer's binding to trypsin and IgG. Beads coated with IFN-γ, BSA, trypin, or IgG were incubated with FITC-labeled B1–4, and analyzed by flow cytometry. Random DNA from the initial DNA library pool was used as the control. As presented in Figure 3, aptamer B1–4 generated a significant binding to IFN-γ (Figure 3A), but a relatively weak binding to albumin, trypsin, or IgG (Figure 3B, C & D). The results suggested that, between IFN-γ and albumin, aptamer B1–4 exhibited a binding specificity towards the former. As a comparison, similar experiments were conducted using another aptamer T2, which was the only DNA aptamer described in literature that could bind to IFN-γ [33], [34]. The results showed that while T2 could indeed bind to IFN-γ (Figure 3E), it also bound to albumin to a large degree (Figure 3F). The data indicated that, compared to T2, aptamer B1–4 had lower cross reactivity to albumin and was more specific for binding with IFN-γ.

**Figure 4:**
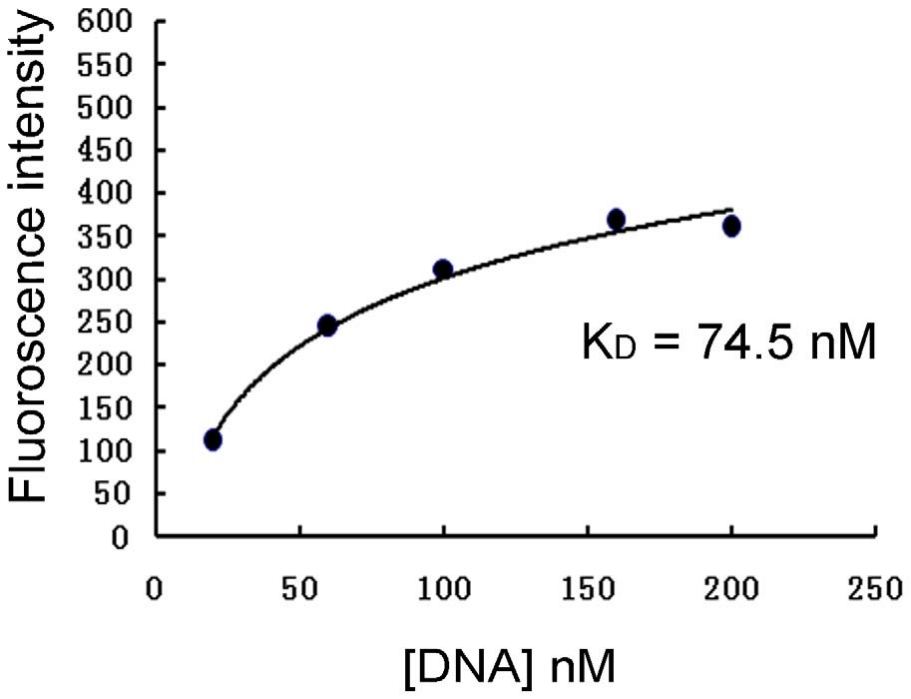
Evaluation of B1–4's affinity to IFN-γ. The affinity between FITC-labeled B1–4 and IFN-γ was quantitatively assayed by plotting the fluorescence against the DNA concentration.

To quantitatively evaluate the binding affinity of aptamer B1–4, IFN-γ-coated beads and blank beads were incubated separately with FITC-labeled B1–4 of various concentrations and analyzed by flow cytometry. Using a non-linear regression analysis, the Kd of aptamer B1–4 for binding with IFN-γ was determined to be 74.5 nM (Figure 4).

### Binding to intracellular IFN-γ by aptamer B1–4

Our previous experiments demonstrated that aptamer B1–4 could bind to IFN-γ coated on magnetic beads. For ICS application, it was important to evaluate whether B1–4 could also bind to the IFN-γ molecules within lymphocytes. Generally, non-immune cells do not produce IFN-γ spontaneously because the gene is suppressed in a stationary status. Only immune cells including dendritic cells, natural killer (NK) cells, or activated CD4+ and CD8+T cells can generate IFN-γ when stimulated by antigens or mitogens. In this study, we used PMA and ionomycin to induce IFN-γ production in lymphocytes. The cells were stained with fluorescence-labeled ligands of antibody or aptamer. Flow cytometry was employed to monitor the bindings of the ligands to intracellular IFN-γ.

**Figure 5:**
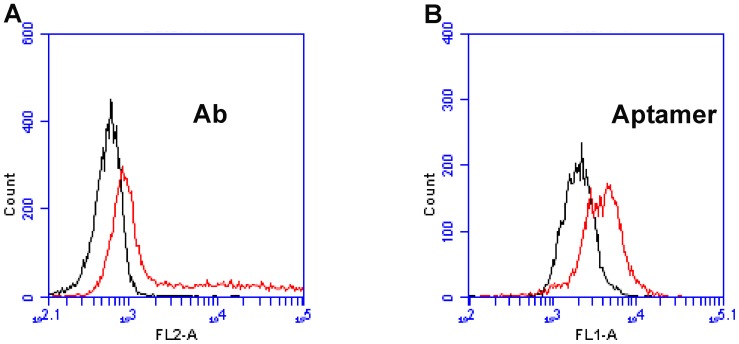
Comparative assessment of bindings to IFN-γ in stimulated or unstimulated lymphocytes by antibody (A) and aptamer B1–4 (B). Red and black curves represent stimulated and unstimulated lymphocytes, respectively.

To determine whether the above stimulation protocol could induce IFN-γ production in lymphocytes, cells stimulated with PMA and ionomycin were incubated with fluorescence-labeled IFN-γ antibody. Non-stimulated cells served as the control. As shown in Figure 5A, the fluorescence of the stimulated cells was significantly stronger than the control, indicating that PMA stimulation triggered the production of IFN-γ that could bind to fluorescence-labeled antibody. To determine whether aptamer B1–4 could also bind to intracellular IFN-γ, FITC-labeled B1–4 was incubated with the stimulated lymphocytes, using the unstimulated cells as the control. As shown in Figure 5B, the fluorescent signal of the stimulated cells increased significantly over the control, indicating that aptamer B1–4 could enter the lymphocytes and bound to the IFN-γ produced by these cells.

### Development of aptamer-based assay of intracellular IFN-γ

**Figure 6:**
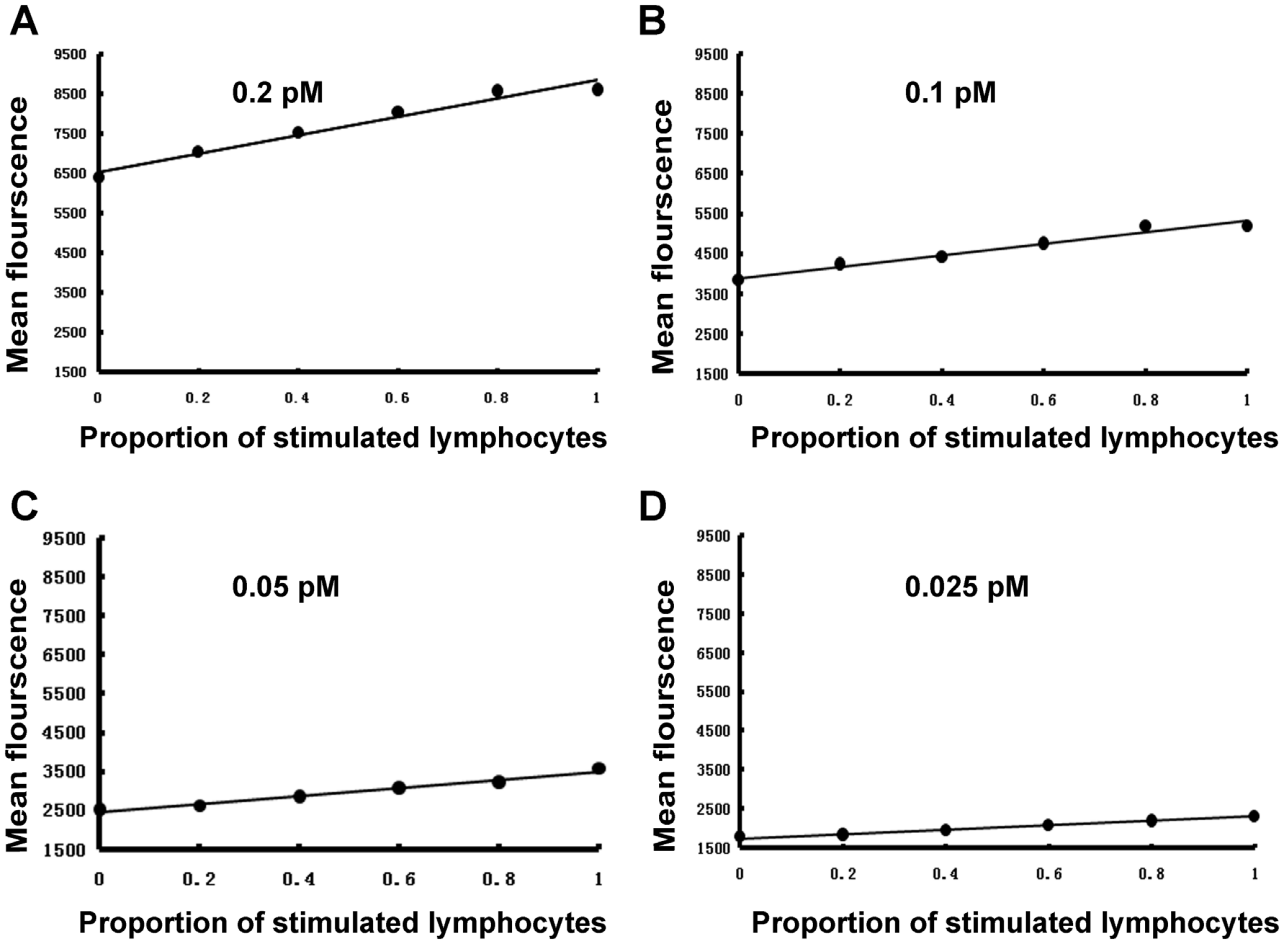
Flow cytometry assessment of the bindings to intracellular IFN-γ by B1–4 of various concentrations. Lymphocytes containing increasing proportions of stimulated cells were incubated with aptamre B1–4 of various concentrations, including 0.2 pM (A), 0.1 pM (B), 0.05 pM (C), and 0.025 pM (D). The lymphocytes were subsequently analyzed by flow cytometry.

Among a group of lymphocytes, the proportion of cells actively producing IFN-γ is of pivital importance for assessing T cell-mediated immunity. Conventionally, the estimation of intracellular IFN-γ is accomplished by staining a group of permeated lymphocytes with fluorescence-labeled antibody and subsequent flow cytometry analysis. This immunological assay is known as intracellular cytokine staining (ICS) [9], [10]. To investigate whether aptamer B1–4 could be used as a substitute of antibody in ICS of IFN-γ, lymphocytes were first stimulated with PMA and ionomycin to generate IFN-γ [30]. FITC-labeled aptamer B1–4 was incubated with cells containing various proportions of the stimulated lymphocytes. The cells were subsequently analyzed by flow cytometry. As shown in Figure 6A, the average fluorescence intensities were positively correlated with the proportion of the stimulated lymphocytes in the study sample. The results suggested that B1–4 might potentially be used for assessing the proportion of IFN-γ-producing cells within a group of lymphocytes.

To explore the optimal working concentration of the aptamer, B1–4 at concentrations of 0.2 pM, 0.1 pM, 0.05 pM, or 0.025 pM was incubated with cells containing various proportions of the stimulated lymphocytes, and the average fluorescence of the cells was measured by flow cytometry. As shown in Figure 6, a positive correlation was observed at each concentration of B1–4. While an aptamer concentration of 0.2 pM was associated with more background fluorescence, concentration of 0.025 pM resulted in a slope value that was very low. The aptamer concentration of 0.05 pM generated a moderate slope with limited background fluorescence. As a result, 0.05 pM was chosen as the working concentration of the aptamer in subsequent experiments.

**Figure 7:**
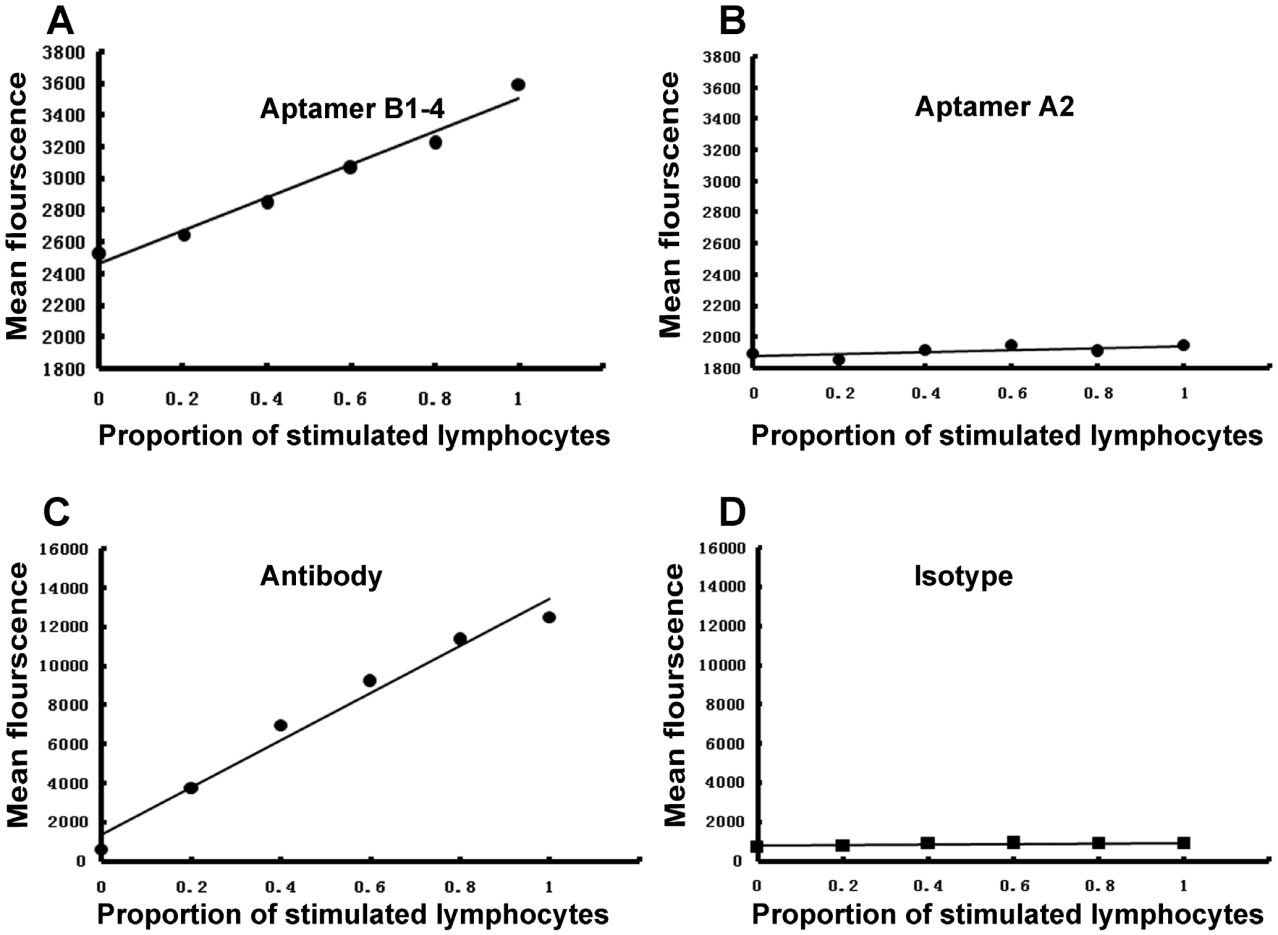
Flow cytometry assessments of the bindings to intracellular IFN-γ by aptamers and monoclone antibodies. A&B, lymphocytes containing increasing proportions of PMA-stimulated cells were incubated with 0.05 pM of aptamer B1–4 (A) or aptamer A2 (B). The latter aptamer does not bind to IFN-γ. C&D, lymphocytes containing increasing proportions of PMA-stimulated cells were incubated with 0.125 µg IFN-γ monoclone antibody (C) or 2 µg isotype antibody (D).

To investigate whether the observed fluorescence was due to a nonspecific absorption of FITC-labeled DNA by lymphocytes, we directly compared, under the same experimental condition, fluorescent signals generated by B1–4 and another aptamer (A2) incapable of binding with IFN-γ. As shown in Figure 7A and B, the fluorescence generated by B1–4 was positively correlated with the number of PMA-stimulated lymphocytes in the study sample, whereas no such correlation was observed with aptamer A2. The results indicated that the high fluorescence observed in stimulated lymphocytes was mainly due to the binding of IFN-γ aptamer, but not the nonspecific absorption of DNA by the cells. As a comparison, parallel experiments were conducted with IFN-γ antibody, using isotype antibody as the control. As show in Figure 7C and D, IFN-γ antibody generated a fluorescence pattern similar to that produced by B1–4, suggesting that aptamer B1–4 might serve as a substitute of antibody in ICS assay of IFN-γ. Nevertheless, it should be noted that the baseline binding of B1–4 was higher than that of the IFN-γ antibody (Figure 7A and C). However, this baseline binding of B1–4 did not obscure the signal of interest, since the signal-to-noise ratio (SNR) was still high enough to reveal the positive correlation between the fluorescence and the number of IFN-γ-producing lymphocytes in the study sample.

## Discussion

The level of IFN-γ provides diagnostic information about infection and the ability of the body to mount an immune response [1]. To our knowledge, this study is the first attempt to use aptamer for assay of intracellular IFN-γ in lymphocytes. We developed a novel 59-mer DNA aptamer B1–4 that could bind to both natural and paraformaldehyde-fixed IFN-γ (Figure 2). The aptamer had minimal cross-reactivity to albumin (Figure 3), and could bind to IFN-γ with a Kd of 74.5 nM (Figure 4). B1–4 was able to enter paraformaldehyde-fixed lymphocytes and bound to the IFN-γ generated by these cells (Figure 5). When a group of cells were stained with FITC-labeled B1–4, the average fluorescence of the cells was positively correlated with the proportion of IFN-γ-producing lymphocytes in the sample (Figure 6). As a result, a standard curve could be established for estimating the number of IFN-γ-producing cells in a given group of lymphocytes (Figure 7).

Antibodies, as the most popular class of molecules for target recognition in a wide range of applications, have been used for more than thirty years. Aptamer may serve as a substitute for antibody and overcome some of the weaknesses associated with antibody-based assay. First, it is well known that proteins are easily denatured and lose their tertiary structure at high temperatures, while oligonucleotides are more thermally stable and can maintain their structures over repeated cycles of denaturation/renaturation[35]. Second, the performance of the antibody heavily depends on the production batch. Aptamers, on the contrary, can be synthesized with great accuracy and reproducibility in large quantity by chemical reactions[36]. For the purpose of ICS assay, aptamers possibly also have advantages. Specifically, aptamer is smaller than antibody, and may readily enter permeated lymphocytes to stain the target cytokine. As a result, the working aptamer concentration required to stain the intracellular target can be quite low, and the cost of the aptamer-based ICS assay may be drastically reduced. Indeed, the amount of aptamer for staining a sample of 10^5^ cells in this study was only 0.05 pM. This means that aptamers generated by a 20-pmol PCR can stain about 400 samples containing 10^5^ cells each, with the potential to significantly reduce the cost of ICS assay.

ICS is the only immunological technique that allows simultaneous determination of both the function and the phenotype of T cells. It is a flow cytometric technique consisting of culturing cytokine-producing cells in the presence of a protein secretion inhibitor, followed by fixation, permeabilization, and staining of intracellular cytokines with a fluorescence-labeled ligand. A prerequisite for implementation of ICS is that the ligand must be capable of binding with paraformaldhyde-fixed target. Because protein structure might change after fixation, and our goal was using aptamer to detect the intracellular IFN-γ in fixed cells, both natural and paraformaldehyde-fixed IFN-γ were used as the targets during the aptamer screening process. This screening strategy was adopted to facilitate the selection of aptamers that could bind to both targets. Nevertheless, we found that most screened aptamers could only bind to one target (data not shown). However, some aptamers did show a binding preference for both targets. Among these aptamers, we identified aptamer B1-4, which could bind to both natural and paraformaldhyde-fixed IFN-γ. This aptamer was suitable for staining IFN-γ in fixed cells and therefore employed in this study.

Previous studies have shown that IFN-γ aptamer can be successfully used as probes in aptamer-based biosensors for assay of IFN-γ. Yan et al developed a aptasensor for label-free electrochemical determination of IFN-γ by taking advantage of ultrahigh electron transfer ability of graphene and DNase I-assisted target recycling signal amplification strategy [25]. Tuleuova et al designed a fluorescence resonance energy transfer (FRET)-based aptamer beacon for monitoring of IFN-γ [26], [27]. Min et al developed a simple and label-free method to detect IFN-γ using aptamer-based electrochemical impedance spectroscopy[28]. So far, however, aptamers have not been explored as ligands for intracellular staining of IFN-γ. In order to implement ICS assay of IFN-γ, the aptamer must have the capability to bind with paraformaldehyde-fixed IFN-γ. For this purpose, we developed a novel IFN-γ aptamer that could recognize both the untreated and the paraformaldhyde-fixed IFN-γ molecules (Figure 2). Moreover, this aptamer was found capable of staining the intracellular IFN-γ in lymphocytes and generating fluorescent signals (Figure 7A & B).

Aptamer needs to possess certain binding specificity and selectivity, in order to have potential in practical applications. Aptamer B1-4 demonstrated a relatively good binding preference for IFN-γ, since it showed a robust binding to PMA-stimulated lymphocytes, but not the unstimulated cells (Figure 5). In this study, we also tested the specificity of aptamer B1–4 by evaluating its binding to albumin, which is the most abundant protein in plasma. As shown in Figures 3, B1–4 could bind to IFN-γ with minimal cross-reactivity to albumin, indicating that aptamer B1–4 had a binding preference for IFN-γ over albumin. Interestingly, the only other DNA aptamer for IFN-γ reported in literature (known as aptamer T2) showed strong binding to both IFN-γ and albumin (Figure 3), for reasons that were not entirely clear. One possibility was that the relatively larger size of B1–4 (59-mer for B1–4 vs. 26-mer for T2) potentially permitted more complexity in its binding function. Nevertheless, aptamer B1–4 also showed some signs of non-specific binding. Notably, when B1–4 was used to stain intracellular IFN-γ, the baseline fluorescence was higher than that of the antibody (Figure 7A and C), suggesting that there might be some non-specific binding to other cellular structures by the aptamer. Although this non-specific binding did not obscure the positive correlation between the fluorescence signal and the proportion of IFN-γ-producing lymphocytes, future research should focus on optimization of the aptamer, reduction of the non-specific binding, or selection of better aptamers with superior binding specificity.

In summary, a novel DNA aptamer for IFN-γ was identified in this study. It can bind to both natural and paraformaldehyde-fixed IFN-γ, enter permeated lymphocytes, and be utilized for estimating the number of IFN-γ-producing cells within a group of lymphocytes. This aptamer provides a novel approach for evaluation of intracellular IFN-γ generated by lymphocytes, and may be employed to develop a more economical ICS assay of IFN-γ with broad application potential.

## References

[1] Boehm U, Klamp T, Groot M, Howard JC (1997) Cellular responses to interferon-gamma. Annu Rev Immunol 15: 749–795. 9143706 10.1146/annurev.immunol.15.1.749

[2] Flynn JL, Chan J, Triebold KJ, Dalton DK, Stewart TA, et al (1993) An essential role for interferon gamma in resistance to Mycobacterium tuberculosis infection. J Exp Med 178: 2249–2254. 7504064 10.1084/jem.178.6.2249 PMC2191274

[3] Pantaleo G, Koup RA (2004) Correlates of immune protection in HIV-1 infection: what we know, what we don't know, what we should know. Nat Med 10: 806–810. 15286782 10.1038/nm0804-806

[4] Apilux A, Ukita Y, Chikae M, Chailapakul O, Takamura Y (2013) Development of automated paper-based devices for sequential multistep sandwich enzyme-linked immunosorbent assays using inkjet printing. Lab Chip 13: 126–135. 23165591 10.1039/c2lc40690j

[5] Yanagisawa N, Dutta D (2012) Enhancement in the sensitivity of microfluidic enzyme-linked immunosorbent assays through analyte preconcentration. Anal Chem 84: 7029–7036. 22861072 10.1021/ac3011632

[6] Dixit CK, Vashist SK, O'Neill FT, O'Reilly B, MacCraith BD, et al (2010) Development of a high sensitivity rapid sandwich ELISA procedure and its comparison with the conventional approach. Anal Chem 82: 7049–7052. 20704394 10.1021/ac101339q

[7] Sedgwick JD, Holt PG (1983) A solid-phase immunoenzymatic technique for the enumeration of specific antibody-secreting cells. J Immunol Methods 57: 301–309. 6338123 10.1016/0022-1759(83)90091-1

[8] Czerkinsky CC, Nilsson LA, Nygren H, Ouchterlony O, Tarkowski A (1983) A solid-phase enzyme-linked immunospot (ELISPOT) assay for enumeration of specific antibody-secreting cells. J Immunol Methods 65: 109–121. 6361139 10.1016/0022-1759(83)90308-3

[9] Reinsmoen NL, Lai CH (2013) Detection of intracellular cytokines. Methods Mol Biol 1034: 353–358. 23775750 10.1007/978-1-62703-493-7_22

[10] Maecker HT, Rinfret A, D'Souza P, Darden J, Roig E, et al (2005) Standardization of cytokine flow cytometry assays. BMC Immunol 6: 13. 15978127 10.1186/1471-2172-6-13 PMC1184077

[11] Nomura L, Maino VC, Maecker HT (2008) Standardization and optimization of multiparameter intracellular cytokine staining. Cytometry A 73: 984–991. 18612990 10.1002/cyto.a.20602

[12] Lovelace P, Maecker HT (2011) Multiparameter intracellular cytokine staining. Methods Mol Biol 699: 165–178. 21116983 10.1007/978-1-61737-950-5_8 PMC4219546

[13] Lamoreaux L, Roederer M, Koup R (2006) Intracellular cytokine optimization and standard operating procedure. Nat Protoc 1: 1507–1516. 17406442 10.1038/nprot.2006.268

[14] Karlsson AC, Martin JN, Younger SR, Bredt BM, Epling L, et al (2003) Comparison of the ELISPOT and cytokine flow cytometry assays for the enumeration of antigen-specific T cells. J Immunol Methods 283: 141–153. 14659906 10.1016/j.jim.2003.09.001

[15] Cox JH, Ferrari G, Janetzki S (2006) Measurement of cytokine release at the single cell level using the ELISPOT assay. Methods 38: 274–282. 16473524 10.1016/j.ymeth.2005.11.006

[16] Navani NK, Li Y (2006) Nucleic acid aptamers and enzymes as sensors. Curr Opin Chem Biol 10: 272–281. 16678470 10.1016/j.cbpa.2006.04.003

[17] Ellington AD, Szostak JW (1990) In vitro selection of RNA molecules that bind specific ligands. Nature 346: 818–822. 1697402 10.1038/346818a0

[18] Tuerk C, Gold L (1990) Systematic evolution of ligands by exponential enrichment: RNA ligands to bacteriophage T4 DNA polymerase. Science 249: 505–510. 2200121 10.1126/science.2200121

[19] Nimjee SM, Rusconi CP, Sullenger BA (2005) Aptamers: an emerging class of therapeutics. Annu Rev Med 56: 555–583. 15660527 10.1146/annurev.med.56.062904.144915

[20] Proske D, Blank M, Buhmann R, Resch A (2005) Aptamers—basic research, drug development, and clinical applications. Appl Microbiol Biotechnol 69: 367–374. 16283295 10.1007/s00253-005-0193-5

[21] Carothers JM, Oestreich SC, Szostak JW (2006) Aptamers selected for higher-affinity binding are not more specific for the target ligand. J Am Chem Soc 128: 7929–7937. 16771507 10.1021/ja060952q PMC4287982

[22] Han K, Liang Z, Zhou N (2010) Design strategies for aptamer-based biosensors. Sensors (Basel) 10: 4541–4557. 22399891 10.3390/s100504541 PMC3292130

[23] Yang CJ, Jockusch S, Vicens M, Turro NJ, Tan W (2005) Light-switching excimer probes for rapid protein monitoring in complex biological fluids. Proc Natl Acad Sci U S A 102: 17278–17283. 16301535 10.1073/pnas.0508821102 PMC1297691

[24] Tombelli S, Minunni M, Luzi E, Mascini M (2005) Aptamer-based biosensors for the detection of HIV-1 Tat protein. Bioelectrochemistry 67: 135–141. 16027048 10.1016/j.bioelechem.2004.04.011

[25] Yan G, Wang Y, He X, Wang K, Liu J, et al (2013) A highly sensitive label-free electrochemical aptasensor for interferon-gamma detection based on graphene controlled assembly and nuclease cleavage-assisted target recycling amplification. Biosens Bioelectron 44: 57–63. 23391707 10.1016/j.bios.2013.01.010

[26] Tuleuova N, Revzin A (2010) Micropatterning of Aptamer Beacons to Create Cytokine-Sensing Surfaces. Cell Mol Bioeng 3: 337–344. 21170394 10.1007/s12195-010-0148-5 PMC2991185

[27] Tuleuova N, Jones CN, Yan J, Ramanculov E, Yokobayashi Y, et al (2010) Development of an aptamer beacon for detection of interferon-gamma. Anal Chem 82: 1851–1857. 20121141 10.1021/ac9025237

[28] Min K, Cho M, Han SY, Shim YB, Ku J, et al (2008) A simple and direct electrochemical detection of interferon-gamma using its RNA and DNA aptamers. Biosens Bioelectron 23: 1819–1824. 18406597 10.1016/j.bios.2008.02.021

[29] Davis KA, Abrams B, Lin Y, Jayasena SD (1996) Use of a high affinity DNA ligand in flow cytometry. Nucleic Acids Res 24: 702–706. 8604313 10.1093/nar/24.4.702 PMC145686

[30] Nomura LE, Walker JM, Maecker HT (2000) Optimization of whole blood antigen-specific cytokine assays for CD4(+) T cells. Cytometry 40: 60–68. 10754518 10.1002/(sici)1097-0320(20000501)40:1<60::aid-cyto8>3.0.co;2-j

[31] Picker LJ, Singh MK, Zdraveski Z, Treer JR, Waldrop SL, et al (1995) Direct demonstration of cytokine synthesis heterogeneity among human memory/effector T cells by flow cytometry. Blood 86: 1408–1419. 7632949

[32] Nylander S, Kalies I (1999) Brefeldin A, but not monensin, completely blocks CD69 expression on mouse lymphocytes: efficacy of inhibitors of protein secretion in protocols for intracellular cytokine staining by flow cytometry. J Immunol Methods 224: 69–76. 10357208 10.1016/s0022-1759(99)00010-1

[33] Lee PP, Ramanathan M, Hunt CA, Garovoy MR (1996) An oligonucleotide blocks interferon-gamma signal transduction. Transplantation 62: 1297–1301. 8932275 10.1097/00007890-199611150-00021

[34] Balasubramanian V, Nguyen LT, Balasubramanian SV, Ramanathan M (1998) Interferon-gamma-inhibitory oligodeoxynucleotides alter the conformation of interferon-gamma. Mol Pharmacol 53: 926–932. 9584220

[35] Mascini M (2008) Aptamers and their applications. Anal Bioanal Chem 390: 987–988. 18193207 10.1007/s00216-007-1769-y

[36] Jayasena SD (1999) Aptamers: an emerging class of molecules that rival antibodies in diagnostics. Clin Chem 45: 1628–1650. 10471678

